# Determining the Level of Reaction to a Moving Object in Combat Sports Athletes Using a Composite Index and Machine Learning: A Cross-Sectional Methodological Study

**DOI:** 10.3390/jfmk11040431

**Published:** 2026-10-18

**Authors:** Vyacheslav Romanenko, Yrui Tropin, Rashid Matkarimov, Georgiy Korobeynikov, Fikrat Kerimov, Lesia Korobeinikova, Shukurjon Gaziev, Olha Zadorozhna

**Affiliations:** 1 Department of Martial Arts, Kharkiv State Academy of Physical Culture, 61058 Kharkiv, Ukraine; slavaromash@gmail.com (V.R.); tropin.yurij@gmail.com (Y.T.); 2 Department of Theory and Methodology of Physical Education, Uzbek State University of Physical Education and Sport, Chirchiq 111700, Uzbekistan; rector@jtsu.uz (R.M.); fikrat_kerimov@mail.ru (F.K.); shukurjon.gaziyev@mail.ru (S.G.); 3 Department Combat Sport, National University of Ukraine on Physical Education and Sport, 03150 Kyiv, Ukraine; korlesia.66@gmail.com; 4 Department of Sports Psychology and Pedagogy, Uzbek State University of Physical Education and Sport, Chirchiq 111700, Uzbekistan; 5 Institute of Physical Education and Sport, Technical University in Zvolen, 96001 Zvolen, Slovakia; ozadorozhna@ukr.net; 6 Department of Olympic and Professional Sports, Kherson State University, 76018 Ivano-Frankivsk, Ukraine

**Keywords:** sensorimotor control, temporal accuracy, response stability, anticipatory timing, psychophysiological assessment, digital monitoring, Core ML

## Abstract

**Background.** Reaction to a moving object (RMO) is an important psychophysiological function in combat sports, supporting situational perception, anticipation of an opponent’s movements, and timely technical and tactical actions. **Objectives.** This study aimed to assess this function in combat sports athletes using a composite index and to develop a machine learning model for automated classification. **Methods.** The study included 312 combat sports athletes aged 10–25 years, approximately 10% of whom were female, representing five combat-sport disciplines and a broad range of sports qualifications from beginners to highly qualified athletes. Reaction to a moving object was assessed using the specialized “Reaction RMO Pro” test. The RMO_Index was calculated from standardized measures of mean absolute temporal deviation and its variability, with higher values indicating better overall performance. The model was trained using stage-specific test indicators and characteristics of premature, delayed, and accurate responses. **Results.** Mean absolute temporal deviation was 32.44 ± 10.29 ms, and variability was 21.88 ± 6.87 ms. The proportions of premature, delayed, and accurate responses were 48.18 ± 10.18%, 44.66 ± 10.45%, and 7.16 ± 4.40%, respectively. A balanced target variable, RMO_Level_Target, was derived from the RMO_Index: High, 104; Medium, 104; and Low, 104. Within the training-validation dataset, Create ML reported 100% classification accuracy during model development, while the model achieved 91.67% accuracy on the held-out test subset. The F1-scores were 0.93, 0.94, and 0.88 for the High, Low, and Medium classes, respectively. **Conclusions.** The RMO_Index provided a standardized composite measure of response timing accuracy and stability, and enabled the formation of sample-derived High, Medium, and Low performance levels. The Boosted Tree Classifier reproduced these reference levels with 91.67% accuracy in the held-out test subset. These findings support the feasibility of automated classification of RMO test results within the present sample; however, external validation is required before broader practical application.

## 1. Introduction

In modern combat sports, the effectiveness of competitive performance is largely determined not only by the athlete’s level of physical, technical-tactical, and functional preparedness, but also by the ability to quickly perceive the changing situation of a bout, anticipate the opponent’s actions, and implement a motor response in a timely manner [1,2].

Combat sports are characterized by high variability of motor actions, limited time for decision-making, and constant changes in distance, direction of attack, tempo, and rhythm of interaction with the opponent [3,4,5]. Under such conditions, sensorimotor and perceptual-cognitive skills become particularly important, including reaction speed, accuracy in selecting the moment of action, anticipatory ability, and stability of the motor response [6,7]. One important component of sensorimotor preparedness in combat sports athletes is the reaction to a moving object (RMO) [8]. In combat sports practice, this ability is manifested when responding to an opponent’s attacking or defensive actions, changes in distance, the initiation of an attack, movement, and the selection of the optimal moment for a counterattack. Therefore, the reaction to a moving object may be considered an informative psychophysiological indicator that reflects the quality of spatiotemporal anticipation and sensorimotor control in athletes. Recent studies confirm that elite combat sports athletes have advantages in perceptual anticipation compared with lower-level athletes [9,10]. They respond more quickly and accurately to an opponent’s attacking actions. In particular, one study showed that elite combat athletes demonstrate a better ability to anticipate an opponent’s actions, make decisions faster, and focus their attention on a smaller number of the most informative visual cues [11]. This indicates that response effectiveness in combat sports is associated not only with the mechanical speed of the motor response, but also with the quality of the prior analysis of the emerging competitive situation. In the present study, combat sports included taekwondo, karate, judo, Greco-Roman wrestling, and freestyle wrestling. Although these disciplines differ in their technical and tactical characteristics, they all require rapid processing of visual information, anticipation of an opponent’s actions, and timely motor responses under continuously changing conditions. From a neurophysiological perspective, such responses involve the coordinated interaction of visual motion processing, attentional selection, temporal prediction, sensorimotor integration, and motor execution. Therefore, effective reaction to a moving object reflects the integrated functioning of perceptual, cognitive, and sensorimotor processes rather than motor response speed alone.

A key feature of reacting to a moving object is that it combines several interrelated components, such as the perception of movement speed and trajectory, estimation of the time required for the object to reach a certain position, anticipation of the response moment, and execution of the motor action [12]. For combat sports, this is of fundamental importance, since successful performance often depends not on the fastest possible response, but on timely reaction. A premature response may lead to an incorrect technical-tactical decision, whereas a delayed reaction reduces the effectiveness of defense or counterattack.

Studies involving combat sports athletes confirm the feasibility of using computerized methods to assess sensorimotor reactions. In particular, one study substantiated a method for assessing reaction to a moving object in combat sports athletes using computer technologies. The authors emphasize that this form of testing makes it possible to obtain quantitative indicators that characterize athletes’ response patterns under dynamic conditions close to the specific demands of combat sports [8]. The importance of reaction speed and accuracy is also confirmed by studies in various combat sports. Thus, recent studies involving boxers and athletes from Olympic combat sports have shown that visuomotor reaction may vary depending on functional state, chronotype, mental fatigue, and task conditions. This indicates that sensorimotor indicators are sensitive to the athlete’s current state and may be used not only for one-time diagnostics but also for ongoing monitoring of preparedness [13,14]. An athlete’s reaction under competitive conditions is multicomponent and requires comprehensive analysis [15,16]. In combat sports, not only is the ability to react quickly important, but also the ability to identify the most informative cues in the opponent’s movement. Studies of the perception of attacking actions in combat sports demonstrate that athletes must anticipate the target and direction of the opponent’s attack using information about the position of the body, head, limbs, and other biomechanical cues. Under such conditions, reaction effectiveness depends on the integration of visual perception, attention, anticipation, and motor response [17,18].

The traditional presentation of results for the assessment of reaction to a moving object in the form of separate indicators does not always allow for a quick and unambiguous interpretation of the level of its manifestation. Mean absolute temporal deviation, its variability, and the percentages of premature, delayed, and accurate responses reflect different aspects of sensorimotor activity; however, they require generalization for practical use by a coach or researcher. Therefore, the development of an integral Reaction to a Moving Object index (RMO_Index), which combines the key characteristics of the reaction, namely temporal accuracy and stability, is a promising approach. This approach makes it possible to move from an isolated analysis of individual indicators to a comprehensive assessment of the level of reaction to a moving object.

At the same time, the modern development of digital technologies and machine learning methods creates new opportunities for automated interpretation of psychophysiological testing results. In contemporary sport, intelligent data analysis methods are considered one of the promising directions of smart sport training, as they enable the processing of large sets of indicators, the identification of hidden patterns, and support for training decision-making [19,20]. The use of machine learning (ML) models in sports monitoring makes it possible not only to process multidimensional data but also to generate classification decisions that can be applied to the assessment of sports performance, functional state, and risks associated with the training process [21]. In addition, recent reviews emphasize that the combination of sensor technologies, digital platforms, and artificial intelligence algorithms creates conditions for more personalized athlete monitoring and rapid interpretation of the obtained data [22]. For training practice, this is of considerable importance, as it enables rapid interpretation of results, determination of the level of manifestation of the studied function, and formation of an individual athlete profile.

Thus, the study of reaction to a moving object in combat sports athletes is relevant from both theoretical and practical perspectives. Its theoretical significance lies in deepening the understanding of the sensorimotor and perceptual-cognitive mechanisms that ensure the effectiveness of technical-tactical actions under the dynamic conditions of a bout. Its practical significance is associated with the possibility of creating an integral assessment of the manifestation of the studied function and the subsequent use of a machine learning model for the automated determination of the level of reaction to a moving object in combat sports athletes.

The aim of the study was to determine the level of reaction to a moving object in combat sports athletes based on a composite index and to develop a machine learning model for the automated classification of testing results. We hypothesized that: (1) the standardized combination of mean absolute temporal deviation and its variability would provide an integrated measure of RMO performance; and (2) stage-specific temporal-deviation measures and response-direction characteristics would contain sufficient information to reproduce the sample-derived High, Medium, and Low RMO levels using a machine-learning classifier.

## 2. Materials and Methods

*Study Design and Setting*. This cross-sectional methodological study was conducted at the Kharkiv State Academy of Physical Culture, Municipal Youth Sports School “Martial Arts Center”, Comprehensive Youth Sports School No. 2, Slobozhanets Sports Club, and White Bear Sports Club in Kharkiv, Ukraine. The study was designed to develop a composite index of reaction to a moving object and to internally evaluate a machine-learning classification model in combat sports athletes. All psychophysiological assessments were performed using the same Reaction RMO Pro application on an iPad device according to a standardized testing procedure. The reporting of this cross-sectional study was prepared in accordance with the Strengthening the Reporting of Observational Studies in Epidemiology (STROBE) Statement. The completed STROBE checklist is provided as Supplementary Table S1.

*Participants.* The study involved 312 combat sports athletes aged 10–25 years (Mean = 17.5, SD = 4.33 years). Participants were recruited using an availability-based approach from the participating sports institutions during the data-collection period. Athletes who met the inclusion criteria and were available for testing were invited to participate. A total of 312 athletes completed the full Reaction RMO Pro testing procedure and were included in the final analytical sample. The exact number of athletes initially approached and the number who declined participation were not prospectively recorded. Participants were approximately evenly distributed across the age range. Approximately 10% of the sample were female, while the majority were male. The athletes represented five combat-sport disciplines: taekwondo, karate, Greco-Roman wrestling, freestyle wrestling, and judo, with approximately equal representation across disciplines. Sports qualification ranged from beginners to highly qualified athletes, thereby covering a broad spectrum of training experience and competitive preparedness. The five disciplines were analyzed as a pooled sample because the purpose of the study was to develop a common RMO classification framework for combat-sport athletes rather than to compare discipline-specific differences. Accordingly, the resulting RMO_Index thresholds should be interpreted as reference values for the pooled study sample rather than as discipline-specific normative values.

*Study Size.* No formal a priori sample size or statistical power calculation was performed. The study sample comprised 312 combat sports athletes who met the inclusion criteria and completed the full Reaction RMO Pro testing procedure during the data-collection period from late April to July 2026. Because the primary objective was the methodological development of a composite RMO_Index and the internal evaluation of a machine-learning classifier, a conventional power-based sample size calculation was not applied.

*Procedure.* The indicators of reaction to a moving object in combat sports athletes (*n* = 312) were determined using the specialized “Reaction RMO Pro” test for mobile devices running iPadOS [8]. The methodological validity and reliability of the Reaction RMO Pro testing procedure have been previously examined in combat sports athletes [8]. An iPad (9th generation) with a 10.2-inch screen was used as the testing device. All participants were tested using the same physical iPad device and the same standardized Reaction RMO Pro testing procedure, thereby eliminating between-device hardware variation as a potential source of systematic differences in the measured outcomes. No separate hardware-specific latency calibration or numerical latency compensation was performed. The test consisted of three stages. The specific feature of the test exercise was the gradual increase in task complexity. In the first stage, the participants had to respond to a slowly moving object; in the second stage, to a fast-moving object; and in the third stage, to a fast-moving object in the presence of interfering visual stimuli. The number of attempts at each stage was 20, with a total of 60 attempts per test. The test did not measure conventional simple visual reaction time from stimulus onset to motor response. Instead, it recorded the temporal deviation between the participant’s response and the moment at which the moving stimulus reached the target position. Responses occurring within the interval from −3 ms to +3 ms were classified as accurate. This accuracy interval was determined by the discrete movement increment of the stimulus across the device screen. Responses occurring earlier than −3 ms were classified as premature, whereas responses occurring later than +3 ms were classified as delayed. The same −3 ms to +3 ms accuracy interval was applied at all three test stages.

Psychophysiological testing was conducted in the second half of the day. To increase interest and conscious participation, the athletes were informed in advance about the aim of the study, the content of the test tasks, and the practical value of the obtained results for assessing reaction to a moving object. Before the main examination, a practice test was conducted, allowing the participants to become familiar with the task procedure, adapt to the testing conditions, and verify that they understood the instructions correctly. The practice trial was used only for familiarization and was not included in the statistical analysis. Practice effects were not formally quantified in the present study. Before the main testing, all participants confirmed that they understood the task procedure and were ready to perform it according to the instructions.

The study was conducted in accordance with the Declaration of Helsinki and was approved by the Bioethics Commission of the National University of Ukraine on Physical Education and Sport (Protocol No. 4 dated 6 April 2026), prior to participant recruitment and data collection. All participants were informed about the aim of the study, the specifics of the testing procedure, the nature of the data collected, and their right to withdraw from participation at any stage without giving reasons. For underage athletes, written informed consent was obtained from parents or legal representatives, who were present during testing. At the time of the study, all participants were medically cleared to perform the test tasks.

The inclusion criteria were participation in one of the five combat-sport disciplines included in the study and completion of the full Reaction RMO Pro testing procedure. No additional exclusion criteria were applied.

*Sample of Variables.* At each stage of the test, response timing accuracy and its variability were recorded. For consistency with the existing dataset and the implemented Core ML model, the original variable labels containing the abbreviation “RT” were retained; however, these variables do not represent conventional reaction time measured from stimulus onset. Instead, they represent temporal deviation relative to the target moment. The variables RT1_mean, RT2_mean, and RT3_mean represented the mean absolute temporal deviation at stages 1, 2, and 3, respectively, whereas RT1_SD, RT2_SD, and RT3_SD represented the corresponding variability in temporal deviations. Response direction was characterized by the percentages of premature (Premature_%), delayed (Delayed_%), and accurate (Accurate_%) responses, which together amounted to 100%. For model development, these variables were adapted to the CSV format as Premature_pct, Delayed_pct, and Accurate_pct. The integral RMO index was calculated from two overall characteristics: RT_avg, representing the mean absolute temporal deviation across the entire test, and RT_SD, representing the overall variability of temporal deviations, according to the following formula:

RMO_Index = −[z(RT_avg) + z(RT_SD)]

For the calculation of RT_avg, absolute temporal deviations were used; therefore, smaller values indicated more precise temporal matching of the response to the target moment. RT_SD characterized within-test response variability across repeated attempts, with lower values indicating more consistent response timing within the same testing session. This measure was not intended as an estimate of test–retest reliability or between-session stability. Standardization was performed using the following formulas:

z(RT_avg) = [RT_avg − mean(RT_avg)]/SD(RT_avg);

z(RT_SD) = [RT_SD − mean(RT_SD)]/SD(RT_SD)

where z(RT_avg) represents the standardized mean absolute temporal deviation across the entire test, and z(RT_SD) represents the standardized variability of temporal deviations. Standardization placed both components on the same dimensionless scale, and they were assigned equal weight in the RMO_Index. The direction of the resulting index was inverted because lower values of both RT_avg and RT_SD indicate better performance. Consequently, higher RMO_Index values correspond to more accurate and stable response timing. Equal weighting was selected a priori because RT_avg and RT_SD represent complementary dimensions of RMO performance, namely temporal accuracy and within-test response consistency, respectively. Because both components were standardized before aggregation and no independent external criterion was available for estimating optimal weights, no data-driven weighting procedure was applied. To assess the robustness of this assumption, a sensitivity analysis was performed using alternative RT_avg/RT_SD weighting schemes of 25/75, 40/60, 50/50, 60/40, and 75/25. For each weighting scheme, the RMO_Index was recalculated and tertile-based High, Medium, and Low levels were re-derived. Agreement with the original 50/50 classification was assessed using percentage agreement and Cohen’s kappa, and Spearman correlations between the alternative and original index values were calculated.

The percentages of premature, delayed, and accurate responses were not included in the RMO_Index because they characterize the direction and distribution of responses relative to the target moment, whereas the index was specifically designed to summarize two complementary characteristics of temporal performance: the magnitude of temporal deviation and its stability. In addition, Premature_pct, Delayed_pct, and Accurate_pct are mutually dependent proportions that together sum to 100%; their inclusion in the same composite index could therefore introduce redundancy and disproportionate weighting of response-direction characteristics. These variables were retained as complementary descriptive indicators and were used separately as predictors in the machine-learning model. The machine learning model was developed using the Tabular Classification task in Create ML 6.2 within Xcode 27.0.

The target variable, RMO_Level_Target, was derived from the RMO_Index. The levels were determined according to tertiles: High, Medium, and Low. A Boosted Trees algorithm was used as the machine learning method. This algorithm is effective for tabular data and is capable of modeling nonlinear relationships between features. The model parameters (Table 1) were aimed at ensuring a balance between classification accuracy and reducing the risk of overfitting. Limiting the tree depth to 3 levels helped preserve a relatively simple model structure, whereas the use of partial selection of rows and predictors at each iteration increased its generalization ability.

The task of the model was to automatically assign a class according to the existing scaling algorithm. The class distribution in the full sample (n = 312) was balanced: High—n = 104; Medium—n = 104; Low—n = 104. This approach ensured class balance, which is important for training an ML model. Nine predictors were included in the model: mean absolute temporal deviation (RT1_mean, RT2_mean, RT3_mean); temporal-deviation variability (RT1_SD, RT2_SD, RT3_SD); and reaction direction (Premature_pct, Delayed_pct, Accurate_pct). The indicators RT_avg, RT_SD, z_RT_avg, z_RT_SD, and RMO_Index were not included as predictors, as they were used to form the target variable.

*Statistical Analysis and Machine Learning Procedure.* To evaluate the quality of the classification model, a stratified split of the sample into a training-validation subset and a held-out test subset was applied. The training-validation subset included 264 observations, while the held-out test subset included 48 observations. Stratification ensured the same ratio of High, Medium, and Low classes in each part of the sample, which reduced the risk of biased model evaluation associated with class imbalance. In the training-validation subset, each class was represented by 88 observations, whereas in the held-out test subset, each class was represented by 16 observations. The quality of the classification model was assessed using accuracy, precision, recall, and F1-score. Accuracy was used for the overall assessment of the proportion of correctly classified results. Precision characterized the proportion of correct predictions among all cases assigned by the model to a particular class. Recall reflected the model’s ability to identify all observations that actually belonged to the corresponding class. F1-score was used as a generalized metric combining precision and recall and is informative for comparing classification quality across individual classes. As a deterministic baseline, the RMO_Index tertile thresholds were applied directly to assign High, Medium, and Low levels. Because these same thresholds were used to define RMO_Level_Target, this rule-based approach reproduces the reference labels by construction and was used as a benchmark for interpreting the added value of the machine-learning classifier. Additional statistical calculations, such as descriptive statistics and determination of the composite index of reaction to a moving object, were performed in RStudio (version 2026.01.0 + 392). Descriptive statistics are presented as mean (Mean), standard deviation (SD), first and third quartiles (Q1 and Q3), median (Median), and minimum and maximum values (Min and Max). The sampling uncertainty of the RMO_Index tertile cut-offs was evaluated using nonparametric bootstrap resampling with 10,000 iterations. In each bootstrap sample, RT_avg and RT_SD were re-standardized, the RMO_Index was recalculated, and the 33.3rd and 66.7th percentiles were estimated. Bootstrap percentile 95% confidence intervals were obtained from the 2.5th and 97.5th percentiles of the bootstrap distributions. A 95% exact binomial confidence interval was also calculated for classification accuracy on the held-out test subset.

The 264 observations constituted a single training-validation dataset used for model development in Create ML. The validation reported by Create ML was performed internally within this same training-validation dataset and therefore does not represent an additional independent sample of 264 participants. The held-out test subset (n = 48) was kept separate from model development and was used only for the final evaluation of classification performance.

## 3. Results

To characterize the study sample and ensure reproducibility of the obtained results, descriptive statistics for the study participants (n = 312) were calculated and are presented in Table 2.

The analysis of descriptive statistics for the results of the reaction to a moving object test provided a general characterization of the study sample. Across the sample, premature responses accounted for 48.18 ± 10.18% of the trials, delayed responses for 44.66 ± 10.45%, and accurate responses for 7.16 ± 4.40%. Thus, premature and delayed responses were substantially more frequent than accurate responses. These proportions should be interpreted as characteristics of response distribution under the specific Reaction RMO Pro task conditions rather than as direct indicators of athlete ability, because the proportion of accurate responses depends on the predefined −3 to +3 ms accuracy interval.

For a generalized assessment of the testing results, an integral Reaction to a Moving Object index (RMO_Index) was developed. Its calculation was based on two main indicators characterizing the quality of performance in the RMO test: the mean absolute temporal deviation across the entire test (RT_avg) and the overall variability of temporal deviations (RT_SD). The RT_avg indicator reflected the athlete’s response timing accuracy, whereas RT_SD characterized the stability of task performance. Lower values of both indicators indicated a better result, as they corresponded to a more accurate temporal matching and lower response variability to a moving object. Before being combined into a single index, the RT_avg and RT_SD indicators were standardized using z-transformation. This made it possible to convert them into a common dimensionless scale and ensure their correct combination into one integral indicator. Based on the RMO_Index values, three levels of reaction to a moving object were formed: High, Medium, and Low. To ensure class balance, tertile-based division was applied. As a result, the target variable RMO_Level_Target was formed, in which each class was represented by an equal number of observations (Table 3).

Bootstrap analysis indicated sampling uncertainty around the sample-derived tertile thresholds. The 95% bootstrap confidence interval was approximately −0.77 to −0.35 for the lower RMO_Index cut-off (−0.56) and 0.94 to 1.19 for the upper cut-off (1.08). These intervals indicate that the precise numerical boundaries are sample-dependent and should not be interpreted as fixed normative thresholds. Sensitivity analysis showed that the RMO classification remained highly stable across alternative weighting schemes for RT_avg and RT_SD (Supplementary Table S2). Agreement with the original 50/50 classification ranged from 94.9% to 98.1%, with Cohen’s κ ranging from 0.923 to 0.971 and Spearman’s ρ from 0.996 to 0.999. All observed classification changes occurred between adjacent RMO levels, with no direct transitions between the High and Low levels.

After the formation of the integral RMO_Index and the target variable RMO_Level_Target, a machine learning model was developed for the automated classification of the level of reaction to a moving object. The model assigned an athlete’s result to one of three levels: High, Medium, or Low. To build the model, indicators characterizing RMO test performance at individual stages, as well as percentage characteristics of reaction direction, were used. The predictor set included nine variables: RT1_mean, RT1_SD, RT2_mean, RT2_SD, RT3_mean, RT3_SD, Premature_pct, Delayed_pct, and Accurate_pct. These indicators reflected response timing accuracy and variability at each test stage, as well as the ratio of premature, delayed, and accurate reactions. The Boosted Tree Classifier algorithm was used to train the model in the Create ML environment in Xcode. This algorithm was selected due to its ability to work with tabular data, identify nonlinear relationships between predictors, and generate classification decisions based on a set of features. To evaluate classification performance, accuracy, precision, recall, and F1-score were used. Accuracy reflected the overall proportion of correctly classified results. Within the training-validation dataset (n = 264), Create ML reported 100% classification accuracy during model development. When evaluated on the held-out test subset (n = 48), which was not used during model development, the model correctly classified 44 of 48 observations, corresponding to an overall accuracy of 91.67% (95% CI: 80.0–97.7%).

Precision characterized the proportion of correct predictions among all cases that the model assigned to a particular class. Recall showed the model’s ability to identify all observations that actually belonged to the corresponding class. F1-score was used as a generalized indicator that combines precision and recall and allows the quality of classification to be assessed separately for each RMO level (Table 4 and Table 5).

The confusion matrix showed that 44 of 48 observations were correctly classified, corresponding to an overall accuracy of 91.67% (95% exact binomial CI: 80.0–97.7%). Of the 16 High-level cases, 14 were correctly classified, and two were classified as Medium. Of the 16 Medium-level cases, 14 were correctly classified, and two were classified as Low, whereas all 16 Low-level cases were correctly identified. No direct misclassification occurred between the High and Low levels. Thus, all classification errors occurred between adjacent RMO levels, which is consistent with the lower F1-score observed for the Medium class. For comparison, direct application of the RMO_Index tertile thresholds reproduced the reference High, Medium, and Low labels with 100% agreement by construction, because these thresholds were used to define RMO_Level_Target. The Boosted Tree Classifier correctly classified 44 of 48 observations in the held-out test subset (91.67%). Thus, the ML model did not outperform the deterministic threshold rule in classification accuracy. Its role was to reproduce the sample-derived classification from stage-specific multidimensional predictors and to provide class probabilities suitable for automated on-device implementation.

**Figure 1:**
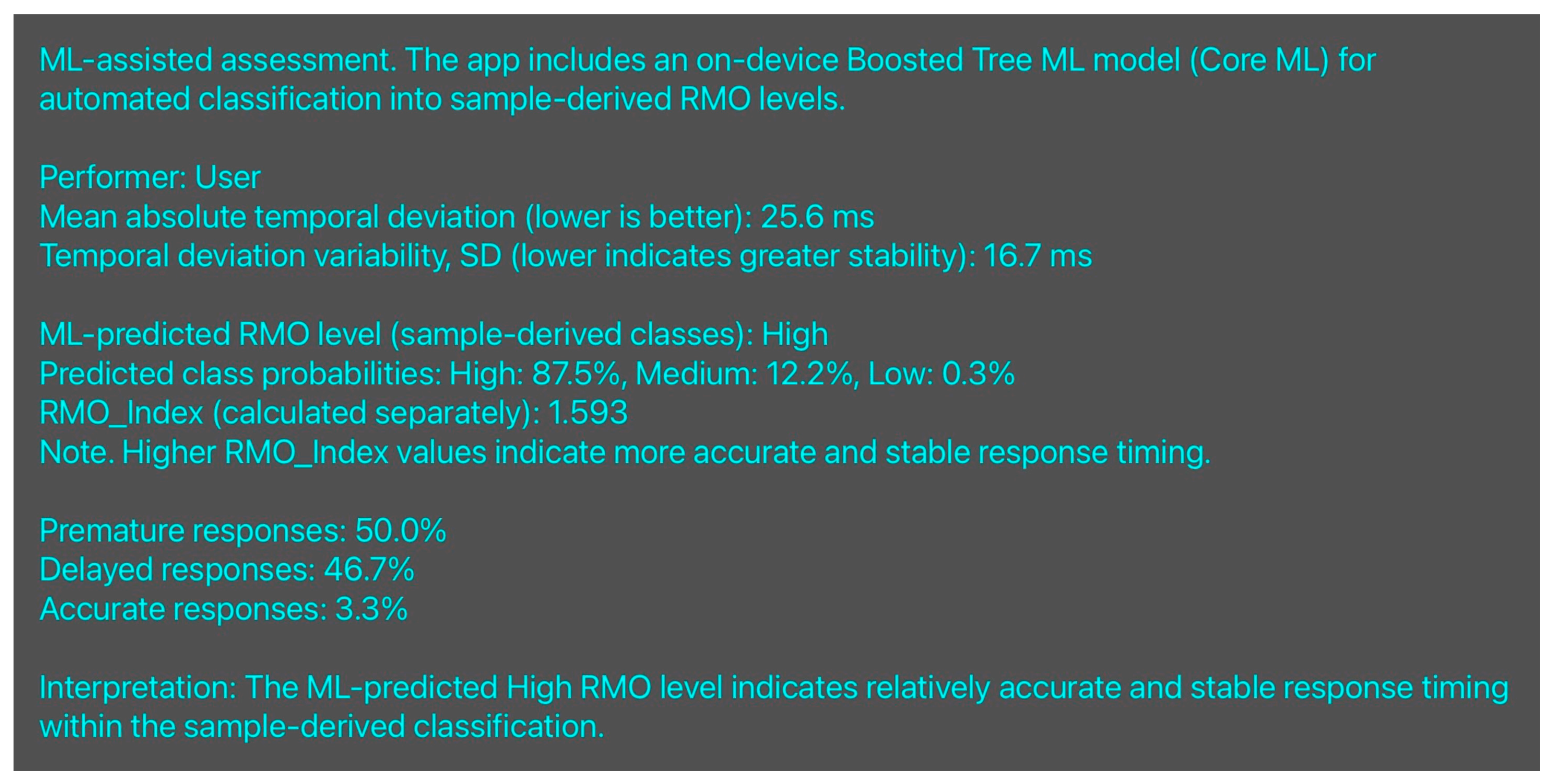
Mobile application screen showing the automated assessment of the level of reaction to a moving object based on a machine learning model.

The trained model was exported in Core ML format, which enabled its subsequent integration into the “Reaction RMO Pro” mobile application on iPadOS (Figure 1).

## 4. Discussion

The results of the present study confirm the feasibility of a comprehensive approach to assessing reaction to a moving object in combat sports athletes. The conditions of competitive activity in combat sports require athletes not only to produce a fast motor response but also to accurately anticipate the moment of interaction with a moving object, maintain reaction stability, and control the timeliness of action execution. This is consistent with data from systematic reviews, which emphasize that combat sports athletes must quickly identify relevant visual cues, anticipate the opponent’s actions, and make decisions under time constraints [11,23]. Therefore, assessing the RMO function only by mean absolute temporal deviation is insufficient, as it does not fully reflect individual characteristics of sensorimotor control.

In the present study, reaction to a moving object was considered an integral characteristic combining accurate and stable response timing. For this reason, the RMO_Index was formed to summarize the results and included standardized values of mean absolute temporal deviation and temporal-deviation variability. This approach made it possible to move from the analysis of separate indicators to a comprehensive assessment of the RMO function level and to form balanced High, Medium, and Low classes for subsequent machine learning. The feasibility of using computerized testing of reaction to a moving object in combat sports athletes is supported by previous studies, which have shown that the RMO method allows quantitative assessment of response characteristics under conditions of gradually increasing task complexity [8]. Across the sample, premature and delayed responses predominated over accurate responses, reflecting the distribution of response timing under the specific conditions of the RMO task. However, these proportions are influenced by the predefined accuracy interval and therefore should not be interpreted independently as direct indicators of athlete ability or response strategy. The predominance of premature or delayed responses may reflect individual response patterns under the specific conditions of the RMO task. However, these proportions are influenced by the predefined accuracy interval and therefore should not be interpreted independently as direct indicators of athlete ability or response strategy.

The obtained results provided a methodological basis for developing a machine learning model that performed automated classification of the level of reaction to a moving object. The classification accuracy observed in the held-out test subset reflects close agreement between the model predictions and the sample-derived High, Medium, and Low RMO levels. The use of machine learning in this context corresponds to current trends in sports analytics, where AI/ML approaches are applied to analyze multidimensional data, support the monitoring of athletes’ preparedness, and assist coaching decision-making. However, researchers emphasize the need for model interpretability, external validation, and cautious use of automated conclusions [24,25].

One of the key results of the study is the development of the integral RMO_Index, which combines two basic components of reaction quality: temporal accuracy and stability. This approach is methodologically appropriate because mean absolute temporal deviation alone does not allow a full characterization of an athlete’s level of sensorimotor control. With the same mean absolute temporal deviation, athletes may differ substantially in response variability, which is important for combat sports, where the effectiveness of an action depends not only on speed but also on the repeatability and controllability of the response. This is consistent with current views on perceptual-cognitive activity in combat sports, according to which an athlete’s success is determined not only by reaction speed but also by the ability to effectively use visual information, anticipate the opponent’s actions, and implement a motor response in a timely manner [11,23,26]. The results of the study also indicate the importance of reaction direction in the RMO test. The proportions of premature and delayed responses provide descriptive information about response direction under the specific RMO task conditions. Because these proportions are partly determined by the predefined accuracy interval, they should not be interpreted as validated indicators of anticipatory, cautious, or other response strategies. Nevertheless, they may reflect different response tendencies that warrant further investigation. In combat sports, anticipatory responding may be relevant because expert athletes make more effective use of advance information about an opponent’s actions and demonstrate advantages in perceptual anticipation compared with lower-level athletes [11]. At the same time, the literature indicates that isolated reaction time is not consistently supported as a predictor of success in combat sports and should therefore be considered together with other perceptual-cognitive characteristics [27].

The division of athletes into High, Medium, and Low levels based on the tertile distribution of the RMO_Index made it possible to move from a purely quantitative assessment to a classification that is more convenient for practical use. This classification format may facilitate interpretation in future sports-monitoring applications, subject to external validation. At the same time, it is important to emphasize that these levels are relative and were formed based on a specific reference sample of combat sports athletes. The Medium level was the most difficult to classify, which is methodologically expected. This class is located between the extreme High and Low levels; therefore, its boundaries are less distinct. Medium-level athletes may have mixed profiles, that is, relatively accurate but variable response timing or, conversely, a stable response timing with greater temporal deviation. For this reason, distinguishing the Medium level is more difficult than identifying the High and Low levels. A similar problem is typical of classification tasks in sports analytics, where intermediate classes often have less pronounced features than extreme categories, and model quality largely depends on the size, balance, and representativeness of the data [28].

Previous studies on the computerized assessment of reaction to a moving object in combat sports athletes also emphasize the appropriateness of using quantitative indicators to characterize psychophysiological functions under conditions of gradually increasing task complexity [8]. It is important to note that RT_avg, RT_SD, their standardized values, and RMO_Index were not included in the predictor set because these variables were directly used to define RMO_Level_Target. However, the stage-specific variables RT1_mean-RT3_mean and RT1_SD-RT3_SD were calculated from the same underlying RMO trials and are therefore structurally related to the aggregate measures used to construct the target variable. Thus, although direct target leakage was avoided, the predictors and the target were not fully independent. Accordingly, the 91.67% test accuracy should be interpreted primarily as the model’s ability to reproduce the sample-derived RMO_Index classification from stage-specific test characteristics rather than as independent predictive validation of an external outcome. The use of machine learning for the analysis of sports data corresponds to current trends, as ML approaches make it possible to process multidimensional indicators, identify complex relationships between variables, and support the monitoring of athletes’ preparedness [28,29]. The direct RMO_Index threshold rule remains the simplest and most accurate method for reproducing the sample-derived levels because these levels are defined directly from the index. Accordingly, the ML model should not be viewed as a replacement for, or an improvement over, the deterministic rule in terms of classification accuracy. Its contribution in the present study is primarily methodological and technical: it demonstrates the feasibility of reproducing the same classification from multidimensional stage-specific features and implementing probabilistic classification within an on-device Core ML environment.

Recent studies indicate that machine learning methods are increasingly being used specifically in combat sports to analyze technical-tactical actions, predict performance, assess athletes’ functional state, and support the training process. Studies devoted to sports analytics emphasize that ML algorithms make it possible to identify hidden relationships between athletes’ psychophysiological and technical characteristics that are difficult to detect using traditional statistical methods [19,30]. In particular, recent studies use artificial intelligence for the automated analysis of athletes’ motor actions, prediction of the effectiveness of technical-tactical actions, assessment of the risk of overfatigue, and individualization of training loads [21,31]. In this context, the proposed classification model demonstrates the technical feasibility of using ML to reproduce sample-derived RMO levels from multidimensional stage-specific test features in combat-sport athletes. It is also important that the use of an on-device Core ML model enables the mobile application to operate autonomously without the need to transmit data to external servers, which increases the efficiency of sports monitoring and simplifies the integration of digital technologies into everyday training practice.

The potential practical significance of the obtained results lies in the possibility of using the RMO_Index and the machine-learning model for automated interpretation of RMO test results, subject to external validation. The proposed approach makes it possible not only to determine separate indicators of response timing accuracy and variability, but also to form a generalized level of manifestation of the RMO function. This may be useful for monitoring psychophysiological functions, identifying individual response characteristics, and controlling changes during the training process. The integration of the model into a mobile application creates conditions for the automated interpretation of testing results immediately after its completion. This approach may simplify the use of the RMO test in coaching practice, since the result is presented not only as numerical indicators but also as a classification level—High, Medium, or Low—with corresponding probabilities of belonging to each class. At the same time, recent reviews on the use of artificial intelligence in sports science emphasize that such models require transparent description, testing on independent samples, and cautious interpretation of results in the practice of the training process [32,33,34]. The obtained results suggest the potential of this approach for future smart monitoring in combat sports. Unlike the traditional approach, in which the results of psychophysiological testing are analyzed mainly after the examination has been completed, the integration of an ML model into a mobile application allows rapid data interpretation directly during the training process. Future longitudinal studies should determine whether this approach can be used to detect changes in athletes’ sensorimotor performance over time. Recent studies on artificial intelligence in sport emphasize that the combination of mobile platforms, wearable technologies, and ML algorithms forms the basis for the development of next-generation adaptive sports monitoring systems [20,35]. In the context of combat sports, this is especially important, since an athlete’s psychophysiological state may change rapidly under the influence of fatigue, emotional tension, training load intensity, and the specific demands of competitive activity.

At the same time, the study has certain limitations. First, the formed High, Medium, and Low levels are relative, as they are based on the tertile distribution of the integral RMO_Index in a specific sample of 312 combat sports athletes. The bootstrap confidence intervals further indicate sampling uncertainty around the exact numerical cut-offs; therefore, these thresholds should be considered sample-derived reference values rather than fixed normative boundaries. Second, the wide age range and unequal sex distribution of the sample should be considered when interpreting the results. Because the number of athletes initially approached and those who declined participation were not prospectively recorded, the participation rate could not be calculated, which limits the assessment of potential recruitment bias. The model also requires further external validation in independent samples representing different ages, sexes, sports qualification levels, and combat sport disciplines. The need for external validation and data standardization is one of the key methodological issues in contemporary AI/ML research in sport [28,32]. Future studies should examine age-, sex-, and discipline-specific reference values for the RMO_Index. Third, this study did not examine associations between the RMO levels and independent outcomes such as competitive performance, technical-tactical effectiveness, fatigue, injury risk, or training outcomes. Therefore, the obtained levels should be interpreted only as sample-derived categories of RMO test performance. Although within-session response variability was quantified by RT_SD, practice effects were not formally evaluated, and repeated-session measurements were not obtained. Therefore, test–retest reliability of the RMO_Index, RT_avg, and RT_SD could not be evaluated using ICCs, and RT_SD should not be interpreted as evidence of between-session stability. The held-out test subset was relatively small (n = 48; 16 observations per class); therefore, the reported classification metrics should be interpreted cautiously and require confirmation in a larger independent sample. A further technical limitation is that device-specific display and touch latency were not independently measured or compensated for; therefore, the absolute temporal-deviation values may include a constant hardware-related component. In addition, the proportions of premature, delayed, and accurate responses are task-dependent and partly determined by the predefined ±3 ms accuracy criterion; consequently, they should not be interpreted as independent measures of athlete ability.

The prospects for further research include expanding the sample, testing the stability of the integral RMO_Index in different groups of athletes, and conducting external validation of the ML model. Future studies should specifically examine the test–retest reliability of the RMO_Index, RT_avg, and RT_SD using intraclass correlation coefficients with 95% confidence intervals. The present study was not designed or powered for subgroup-specific validation; therefore, the pooled RMO_Index cut-offs should not be interpreted as age-, sex-, or discipline-specific normative thresholds. Dedicated validation studies with adequately sized and balanced subgroups are required to determine whether these cut-offs perform similarly across demographic and combat-sport groups. It would also be appropriate to compare different machine learning algorithms for classifying the level of reaction to a moving object, including Random Forest, Support Vector Machine, Neural Network, and other approaches to tabular classification. A separate promising direction is the study of the relationship between the integral RMO_Index, the High, Medium, and Low levels, and indicators of competitive performance in athletes from different combat sports. This will make it possible to clarify the practical significance of the RMO function for bout effectiveness and to substantiate the use of this indicator in the system of comprehensive monitoring of athletes’ preparedness.

## 5. Conclusions

The study developed a composite RMO_Index based on standardized mean absolute temporal deviation and response variability and used this index to define sample-derived High, Medium, and Low RMO performance levels. Response direction, represented by premature, delayed, and accurate responses, was analyzed separately and used as additional information in the machine-learning model. The Boosted Tree Classifier reproduced the sample-derived RMO levels with 91.67% accuracy in the held-out test subset, with higher classification performance for the Low level than for the High and Medium levels. These findings demonstrate the feasibility of automated classification of RMO test results within the present sample but do not establish external predictive validity. The High, Medium, and Low levels should not be interpreted as universal normative categories or as validated indicators of competitive performance, fatigue, injury risk, or training outcomes. External validation in independent samples and evaluation against independent performance criteria are required before broader practical application. Integration into Core ML demonstrates the technical feasibility of on-device classification but does not by itself establish practical utility in sports monitoring.

Further research should focus on external validation of the model in independent samples of athletes of different ages, qualification levels, and combat sport disciplines, as well as on studying the relationship between the RMO_Index and the High, Medium, Low levels with indicators of competitive performance.

## Figures and Tables

**Table 1:** Parameters of the Boosted Tree Classifier model.

Parameters	Value
Algorithm	Boosted Tree
Max Iterations	100
Max Depth	3
Min Loss Reduction	0
Min Child Weight	0.1
Row Subsample Ratio	0.8
Column Subsample Ratio	0.8
Step Size	0.1

**Table 2:** Results of psychophysiological testing of combat sports athletes (n = 312).

Parameters	Mean	SD	Median	Q1	Q3	Min	Max
RT_avg, ms	32.44	10.29	30.51	25.25	37.69	13.16	75.06
RT_SD, ms	21.88	6.87	20.64	17.23	25.51	8.73	51.62
RT1_mean, ms	33.35	13.42	32.06	24.48	40.03	8.12	84.56
RT1_SD, ms	22.32	8.99	21.46	16.39	26.52	3.68	60.50
RT2_mean, ms	31.20	12.03	28.68	22.49	37.77	6.99	78.94
RT2_SD, ms	21.17	8.62	19.35	14.89	25.66	4.94	51.10
RT3_mean, ms	32.76	12.50	30.60	24.11	39.70	5.30	79.52
RT3_SD, ms	22.15	8.81	20.77	16.14	26.50	3.10	61.23
Premature, %	48.18	10.18	46.70	41.70	53.30	20.00	86.70
Delayed, %	44.66	10.45	45.00	38.30	50.00	8.30	78.30
Accurate, %	7.16	4.40	6.70	3.30	10.00	0.00	25.00

*Note.* RT_avg—mean absolute temporal deviation across the entire test; RT_SD—overall variability of temporal deviations; RT1_mean-RT3_mean—mean absolute temporal deviation at stages 1–3; RT1_SD-RT3_SD—variability of temporal deviations at stages 1–3.

**Table 3:** Distribution of combat sports athletes by levels of reaction to a moving object based on the RMO_Index (n = 312).

RMO Level	RMO_Index Range	n	%	Interpretation
High	>1.08	104	33.3	More accurate and stable response timing
Medium	−0.56 to 1.08	104	33.3	Intermediate response timing accuracy and stability
Low	≤−0.56	104	33.3	Greater temporal deviation and/or response variability

**Table 4:** Classification performance of the model by RMO levels on the held-out test subset.

RMO Level	n	Precision	Recall	F1-Score
Medium	16	88%	88%	0.88
Low	16	89%	100%	0.94
High	16	100%	88%	0.93

**Table 5:** Confusion matrix of the Boosted Tree Classifier on the held-out test subset (n = 48).

Reference RMO Level	Predicted High	Predicted Medium	Predicted Low	Total
High	14 (87.5%)	2 (12.5%)	0 (0.0%)	16
Medium	0 (0.0%)	14 (87.5%)	2 (12.5%)	16
Low	0 (0.0%)	0 (0.0%)	16 (100.0%)	16
Total	14	16	18	48

*Note*. Values are presented as n (% within the reference RMO level). Rows represent the reference RMO levels derived from the RMO_Index, and columns represent the levels predicted by the Boosted Tree Classifier.

## Data Availability

The anonymized dataset supporting the findings of this study is publicly available in Zenodo at https://doi.org/10.5281/zenodo.21965166. The R code used for the RMO_Index and additional statistical analyses, the machine-learning model configuration, and the trained Core ML model are provided as the Supplementary Materials accompanying this article.

## Supplementary Materials

The following supporting information can be downloaded at: https://www.mdpi.com/article/10.3390/jfmk11040431/s1. Table S1: STROBE checklist for cross-sectional studies; Table S2: Sensitivity analysis of RMO_Index classification under alternative weighting schemes for RT_avg and RT_SD; RMO_analysis. R: analytical R code; RMO_BoostedTree_Classifier.mlpackage.zip: trained Core ML model.

## Abbreviations

The following abbreviations are used in this manuscript:

RT	Reaction time (legacy variable label representing temporal deviation in the present study)
RMO	Reaction to a moving object
