# Supplementary material for: Determining the Level of Reaction to a Moving Object in Combat Sports Athletes Using a Composite Index and Machine Learning: A Cross-Sectional Methodological Study
Source: J Funct Morphol Kinesiol. 2026 Oct 18;11(4):431. doi: 10.3390/jfmk11040431 (PMC13692411; doi:10.3390/jfmk11040431)
Supplement: Supplementary file 1 [file jfmk-11-00431-s001.zip › jfmk-4610938-supplementary.pdf]

Supplementary Table S1. STROBE Checklist for Cross-Sectional Studies

Manuscript: Determining the Level of Reaction to a Moving Object in Combat Sports Athletes Using a Composite Index and Machine Learning: A Cross-Sectional Methodological Study

Note. The checklist is mapped to manuscript sections rather than final page numbers because pagination may change during revision. The previously pending factual items concerning study size, missing data, participant flow, eligibility criteria, and the data-collection period have now been resolved based on the confirmed study information.

Manuscript section	Item	STROBE reporting requirement (paraphrased)	Location in manuscript	Status / manuscript-specific note
Title and abstract	1a	Identify the study design using a standard term in the title or abstract.	Title; Abstract	Reported in the revised title, which identifies the work as “A Cross-Sectional Methodological Study”.
Title and abstract	1b	Provide a balanced abstract summarizing what was done and the principal findings.	Abstract	Reported; revised structured abstract should retain Background, Objectives, Methods, Results, and Conclusions.
Introduction	2	Explain the scientific background and rationale for the investigation.	Introduction	Reported.
Introduction	3	State the specific objectives and any prespecified hypotheses.	End of Introduction	Reported in the revised Introduction: the study objective is followed by the prespecified hypotheses concerning the composite RMO_Index and ML reproduction of the sample-derived RMO levels.
Methods	4	Present the key elements of the study design early in the Methods.	Section 2.1 Study Design and Setting	Reported in Section 2.1 as a cross-sectional methodological study.
Methods	5	Describe the study setting, locations, and relevant data-collection period.	Section 2.1 Study Design and Setting	Reported in Section 2.1: multiple sports settings in Kharkiv, Ukraine, including youth sports schools, sports clubs, and the Kharkiv State Academy of Physical Culture. Data collection was conducted from late April to July 2026.
Methods	6	Describe eligibility criteria and the sources/methods used to select participants.	Section 2.2 Participants; Procedure	Resolved. Eligibility required participation in one of the combat-sport disciplines included in the study and completion of the full Reaction RMO Pro testing procedure. No additional exclusion criteria were applied. Participants were recruited across the study settings described in Section 2.1.
Methods	7	Clearly define the outcome, predictors, and other variables used in the analysis.	Sample of Variables	Reported: RMO_Index, RMO_Level_Target, stage-specific temporal-deviation measures, variability measures, and premature/delayed/accurate response percentages.
Methods	8	For each variable, describe the data source and measurement procedure; explain whether measurement was comparable across participants/groups.	Procedure; Sample of Variables	Reported. Same Reaction RMO Pro procedure, same iPad-based testing approach, three stages, 20 attempts per stage, and common ±3 ms accuracy interval should be stated as applied to all stages.
Methods	9	Describe steps taken to reduce or address potential sources of bias.	Procedure; Statistical Analysis and Machine Learning Procedure	Reported in part: standardized device/procedure, practice test, common testing instructions, stratified class split. Consider explicitly noting these as bias-control measures.
Methods	10	Explain how the study size was determined.	Participants / Study size statement	Resolved. No formal a priori sample-size calculation was performed. The study sample comprised 312 combat sports athletes who met the eligibility criteria and completed the full testing procedure during the data-collection period.
Methods	11	Explain how quantitative variables were handled, including any categorization and the reason for chosen cut-points.	Sample of Variables; Statistical Analysis; Table 3	Reported: z-standardization, equal weighting in RMO_Index, tertile-based High/Medium/Low categories, and category boundaries.
Methods	12a	Describe the statistical and analytical methods used.	Statistical Analysis and Machine Learning Procedure	Reported: descriptive statistics; stratified development/test split; accuracy, precision, recall, F1-score; Boosted Tree classifier; RStudio and Create ML.

Manuscript section	Item	STROBE reporting requirement (paraphrased)	Location in manuscript	Status / manuscript-specific note
Methods	12b	Describe subgroup or interaction analyses, if performed.	Statistical Analysis and Machine Learning Procedure	Not applicable: no subgroup or interaction analyses were performed for the primary study objective.
Methods	12c	Explain how missing data were handled.	Statistical Analysis and Machine Learning Procedure	Resolved. There were no missing data for the variables included in the analysis; therefore, all 312 observations were used in the statistical and machine-learning analyses.
Methods	12d	If relevant, describe analytical methods that account for the sampling strategy.	Statistical Analysis and Machine Learning Procedure	Not applicable to complex survey sampling. The class-stratified train/test split is reported separately.
Methods	12e	Describe sensitivity analyses, if performed.	Statistical Analysis and Machine Learning Procedure	Not applicable: no sensitivity analyses were reported.
Results	13a	Report participant numbers at each stage from recruitment through analysis.	Participant flow / proposed flow diagram	Resolved. A total of 312 combat sports athletes were included; all 312 completed the full Reaction RMO Pro test and all 312 were included in the final analysis. No participants were excluded.
Results	13b	Provide reasons for non-participation or exclusion at each stage.	Participant flow / proposed flow diagram	Resolved. No participants were excluded from the final analysis; therefore, there were no exclusion or non-participation reasons to report after enrolment.
Results	13c	Consider presenting participant flow in a diagram.	Proposed participant flow figure	Resolved for participant flow: 312 athletes were included, 312 completed testing, 0 were excluded, and 312 were analysed. This information can be presented in the study flow diagram requested by Reviewer 2.
Results	14a	Present participant characteristics relevant to interpretation of the study.	Proposed participant characteristics table; Participants	Reported/confirmed: n = 312; age 10–25 years (Mean = 17.5, SD = 4.33), with participants approximately evenly distributed across the age range; approximately 10% female; five combat-sport disciplines with approximately equal representation; sports qualification ranging from beginners to highly qualified athletes.
Results	14b	Report the number of participants with missing data for variables of interest.	Participant characteristics table / Results	Resolved. No participant had missing data for the variables included in the analysis (missing observations = 0).
Results	15	Report outcome summary measures for the cross-sectional sample.	Table 2; Table 3; Results	Reported: descriptive RMO measures, response-direction percentages, RMO_Index-derived levels, and class distribution.
Results	16a	Report the principal estimates/results and their precision when applicable; identify any adjusted analyses.	Tables 4–5; Results	Reported for classification metrics and confusion matrix. No confounder-adjusted inferential estimates were used for the primary classification objective.
Results	16b	When continuous variables are categorized, report the category boundaries.	Table 3	Reported: High > 1.08; Medium -0.56 < RMO ≤ 1.08; Low ≤ −0.56.
Results	16c	Where relevant, translate relative effects into absolute effects over a meaningful period.	-	Not applicable: the study did not estimate relative risks or time-dependent effects.
Results	17	Report any additional analyses such as subgroup, interaction, or sensitivity analyses.	Results	No subgroup/interactions/sensitivity analyses were performed. Additional model evaluation is reported using class-specific metrics and the confusion matrix.
Discussion	18	Summarize the key findings in relation to the study objectives.	Discussion; Conclusions	Reported.
Discussion	19	Discuss important limitations, including potential bias and imprecision.	Limitations paragraph in Discussion	Reported in substantial part: sample-derived thresholds, broad age range, sex imbalance, pooled disciplines, lack of external validation, and absence of competitive-performance validation. Add reliability limitation if not established in prior work.
Discussion	20	Provide a cautious overall interpretation considering objectives, limitations, multiple analyses, and related evidence.	Discussion	Reported after revision: 94% accuracy interpreted as reproduction of sample-derived RMO classification rather than independent external prediction; target–predictor structural relationship acknowledged.

Manuscript section	Item	STROBE reporting requirement (paraphrased)	Location in manuscript	Status / manuscript-specific note
Discussion	21	Discuss the generalizability of the findings to other populations/settings.	Limitations; Future research; Conclusions	Reported: thresholds treated as sample-specific; external validation and age-, sex-, qualification-, and discipline-specific reference values recommended.
Other information	22	State the source of funding and the role of funders.	Funding statement	Reported: "This research received no external funding."

Source note. Checklist structure follows the STROBE reporting recommendations for cross-sectional studies. The recommendation wording has been paraphrased for this manuscript-specific supplementary table.

Suggested manuscript statement: *"The reporting of this cross-sectional study was prepared in accordance with the Strengthening the Reporting of Observational Studies in Epidemiology (STROBE) Statement. The completed STROBE checklist is provided as Supplementary Table S1."*

Supplementary Table S2. Sensitivity analysis of RMO_Index classification under alternative weighting schemes for RT_avg and RT_SD

RT_avg weight	RT_SD weight	Agreement with original 50/50 classification, %	Cohen's κ	Spearman's ρ
0.25	0.75	95.5	0.933	0.997
0.40	0.60	98.1	0.971	0.999
0.50	0.50	100.0	1.000	1.000
0.60	0.40	98.1	0.971	0.999
0.75	0.25	94.9	0.923	0.996

Note. For each weighting scheme, the RMO_Index was recalculated and tertile-based High, Medium, and Low levels were re-derived. Agreement was calculated relative to the original 50/50 weighting scheme. Cohen's κ reflects categorical agreement, and Spearman's ρ reflects the rank correlation between alternative and original RMO_Index values.
